# A Case Report of Spontaneous Coronary Artery Dissection in Pregnancy: A Challenging Diagnosis

**DOI:** 10.7759/cureus.87233

**Published:** 2025-07-03

**Authors:** Zade Zahlan, Anne V Sauber, Cristina Cota Peimbert

**Affiliations:** 1 Internal Medicine, MountainView Hospital, Las Vegas, USA; 2 Cardiology, Touro University, Las Vegas, USA

**Keywords:** atypical spontaneous coronary artery dissection, dissection, internal jugular thrombus, internal jugular venous thrombus, lemierre, lemierre's, lemierre's and lemierre's like syndrome, spontaneous coronary artery dissection, thrombosis in pregnancy, thrombus

## Abstract

Lemierre’s syndrome and spontaneous coronary artery dissection (SCAD) are rare and potentially life-threatening conditions that seldom occur concurrently. Lemierre’s syndrome typically presents as septic thrombophlebitis of the internal jugular vein following an oropharyngeal infection, while SCAD is a non-atherosclerotic tear in the coronary artery wall, often associated with pregnancy. The co-occurrence of these two conditions is exceedingly rare and presents complex diagnostic and management challenges, particularly in pregnant patients. We describe a case of a 36-year-old woman at 22 weeks of gestation who presented with a three-day history of left-sided neck pain, chest pain, and shortness of breath. Initial workup included an electrocardiogram, which showed nonspecific T-wave changes, serial troponin measurements that revealed a rising trend, and a duplex ultrasound of the neck, which revealed an intramural hematoma in the coronary arteries suggestive of SCAD, along with an occlusive thrombus in the left internal jugular vein consistent with Lemierre’s syndrome. This case highlights a rare overlap of SCAD and Lemierre’s syndrome in pregnancy. Hormonal and hemodynamic changes likely contributed to both conditions. Treatment required careful balancing of anticoagulation risks and benefits. To our knowledge, this is one of the first reported cases of concurrent SCAD and Lemierre’s syndrome in a pregnant patient. It underscores the need for vigilant assessment and coordinated care in rare, high-risk vascular presentations during pregnancy.

## Introduction

Lemierre’s syndrome is a rare disease corresponding to an incidence of 3.6 cases per million people per year, affecting young, healthy individuals [1]. It is characterized by metastatic infection that typically starts from the oropharynx and can spread, causing abscesses, septic emboli, and even local thrombosis [2,3]. It is primarily caused by gram-negative anaerobic bacteria, most commonly the *Fusobacterium* species. Lemierre’s syndrome has been shown to have arterial complications, including strokes and involvement of the carotid arteries and the pericardium [3].

Spontaneous coronary artery dissection (SCAD) is a condition that can occur in pregnancy where a tear forms in an epicardial coronary artery without an underlying cause, such as an atherosclerotic plaque rupture, coronary intervention, or trauma [4]. SCAD can affect both men and women, with White women in particular having the highest incidence in their fifth and sixth decades of life. Some risk factors include being in the postpartum state, pregnancy, concomitant fibromuscular dysplasia (FMD), arteriopathies, and stressors [5,6]. The specific scenario of pregnancy and postpartum has been reported in 2% to 18% of cases [7]. This could happen either by direct hormonal effect on the coronary artery wall or possibly as a result of increased physiological hemodynamic stress. It is also important to consider the particular increase in recurrence risk in patients with pregnancy-associated SCAD [8]. Other than pregnancy, there may be an association with other hormonal changes, such as in vitro fertilization, oral contraception, and hormone replacement therapy [9]. Although the etiology is still unclear, the pathophysiology is essentially where the tunica intima of the epicardial coronary artery separates from the adventitia, presumably from some event that disrupts the vessel wall so that a hematoma forms [10].

Diagnosis is suspected with clinical findings such as anginal chest pain accompanied by ECG changes, which can show ST elevation or T wave inversion, and elevated cardiac troponins [11]. Confirmation of diagnosis is made in most patients with coronary angiography, which is further classified into three types based on specific angiography findings [12]. The most common type observed is type 2, which involves a diffuse, long, and smooth stenosis; rather than contrast dye staining of the arterial wall seen in type 1, and focal or tubular stenosis observed in type 3 [12]. At the time of coronary angiography, the coronary artery most frequently affected was the left anterior descending, in up to 40% of cases [8]. Nevertheless, in patients whose diagnosis remains unclear after coronary angiography, intracoronary imaging with optical coherence tomography (OCT) or intravascular ultrasound (IVUS) and repeated coronary angiography after four to six weeks can be considered [13]. We are here to discuss a case of a 36-year-old gravida 3 para 2 (G3P2) female at 22 weeks of gestation with SCAD.

## Case presentation

A 36-year-old female, G3P2, with a history of two prior vaginal deliveries, one of which was preterm at 35 weeks, presented to our service at 22 weeks gestation with worsening focal mandibular swelling and pain for a few days as well as a new onset of chest pain. Her medical history was notable for prediabetes and prior gestational diabetes.

She reported a three-day history of oppressive, muscular-type chest pain that was not associated with exertion. The pain was worsened by deep inspiration and movement, radiated to the neck, and was accompanied by one episode of nausea, vomiting, and palpitations. On the first night of admission, she also experienced mild shortness of breath.

Home medications included vaginal progesterone 200 mg, aspirin 81 mg, and Augmentin. She was initially diagnosed with presumed cellulitis at an urgent care facility for her mandibular swelling, for which Augmentin was prescribed [14]. Aspirin was given by her obstetrician/gynecologist for preeclampsia prophylaxis [15]. She denied smoking, alcohol use, or illicit drug use.

On physical examination, the patient was hemodynamically stable. Notable findings included tenderness and swelling over the left side of the neck. The rest of the physical exam was unremarkable. Vital signs were significant for tachycardia, with a heart rate of 116 beats per minute.

**Figure 1:**
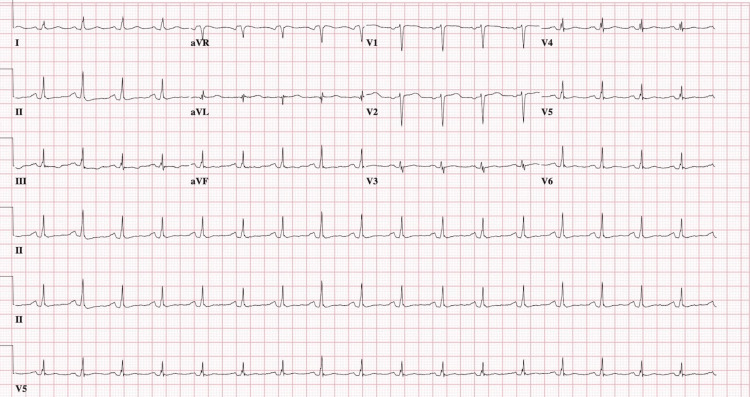
Initial electrocardiogram showing mild T wave depression in lead III.

**Figure 2:**
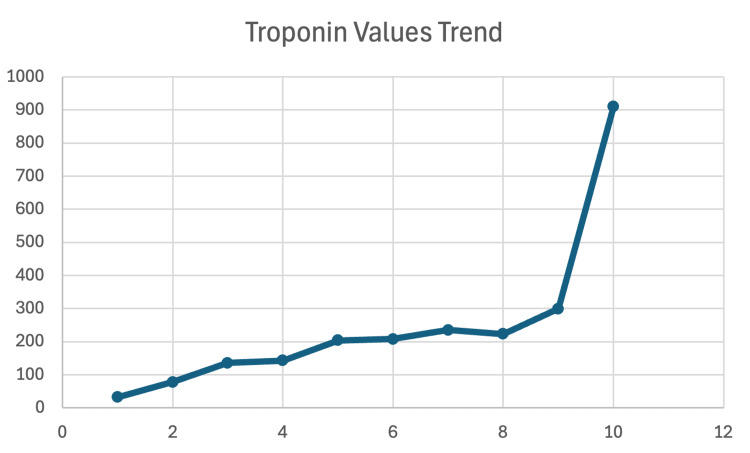
Trend of troponin values (ng/mL). This graph shows the patient's uptrending troponin values over the course of their hospital stay. ng/mL: nanograms per milliliter; X-axis: order of measurement of troponin values; Y-axis: troponin value.

The initial electrocardiogram (ECG) (Figure 1) at presentation showed mild T-wave depression in lead III, with no significant ST-segment elevations or depressions in other leads. There were no axis deviations or rhythm abnormalities, and the tracing demonstrated a normal sinus rhythm. While T-wave changes in isolation, particularly in lead III, are nonspecific, they may be seen in early ischemic changes and should be interpreted cautiously in the setting of suspected SCAD, especially in young females [11]. Serial troponin levels demonstrated a rising trend, with a peak troponin-I level of 911 ng/mL (reference of 0-14ng/mL) (Figure 2), consistent with non-ST elevation myocardial infarction (NSTEMI). This degree of elevation suggests myocardial injury without full-thickness infarction.

Chest X-ray was unremarkable, with no evidence of cardiomegaly, pulmonary edema, or infiltrates. Blood cultures and Gram stain remained negative throughout the initial hospitalization, with no evidence of systemic bacteremia at the time of testing.

**Figure 3:**
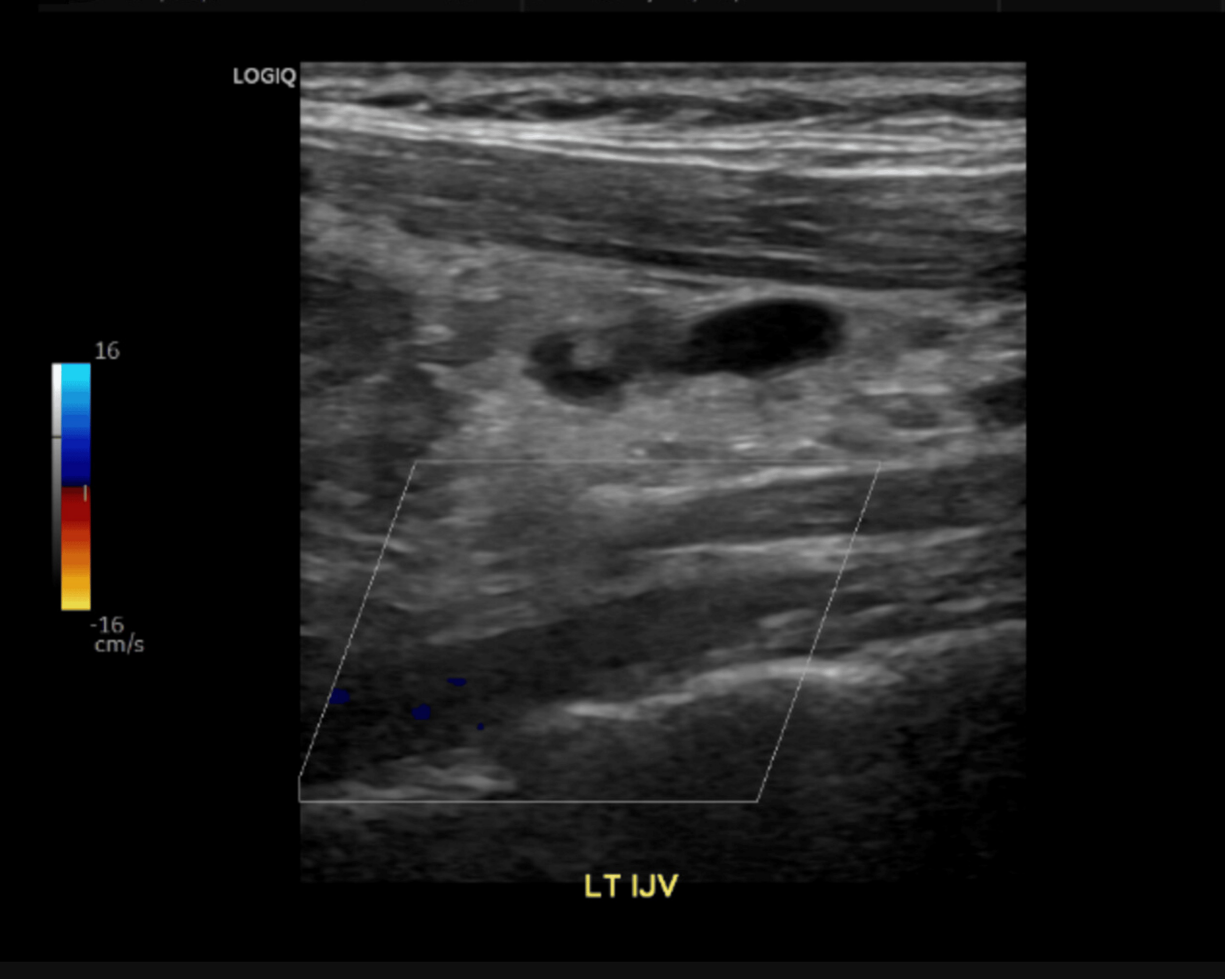
Doppler ultrasound. Upper extremity Doppler ultrasound showing occlusive thrombus in the left internal jugular vein (LT IJV).

A Doppler ultrasound of the upper extremity (Figure 3) revealed an incomplete occlusion of the left internal jugular vein, and in the setting of the patient's symptoms, was consistent with thrombophlebitis, raising suspicion for Lemierre’s syndrome [16]. The left cephalic vein was not visualized, likely due to either technical limitations or thrombotic occlusion, although this could not be definitively confirmed on ultrasound.

Laboratory tests were significant for uptrending serum troponin levels (Figure 2), chest X-ray was without abnormalities, and blood cultures and gram stain showed no growth.

The plan at that time was to start intravenous (IV) ampicillin-sulbactam and enoxaparin, continue the home aspirin and progesterone regimen, consult obstetrics and gynecology for fetal monitoring, and order an echocardiography, computed tomography angiography (CTA), and repeat the troponins [17,18]. We were also monitoring cardiac telemetry and checking the oxygen saturation.

Over the next 48 hours, the patient developed pleuritic chest pain, described as sternal pressure worsened by deep inspiration, along with persistent tachycardia (heart rate: 101-103 bpm). Her neck swelling gradually improved, and the remainder of the physical examination remained unremarkable.

**Figure 4:**
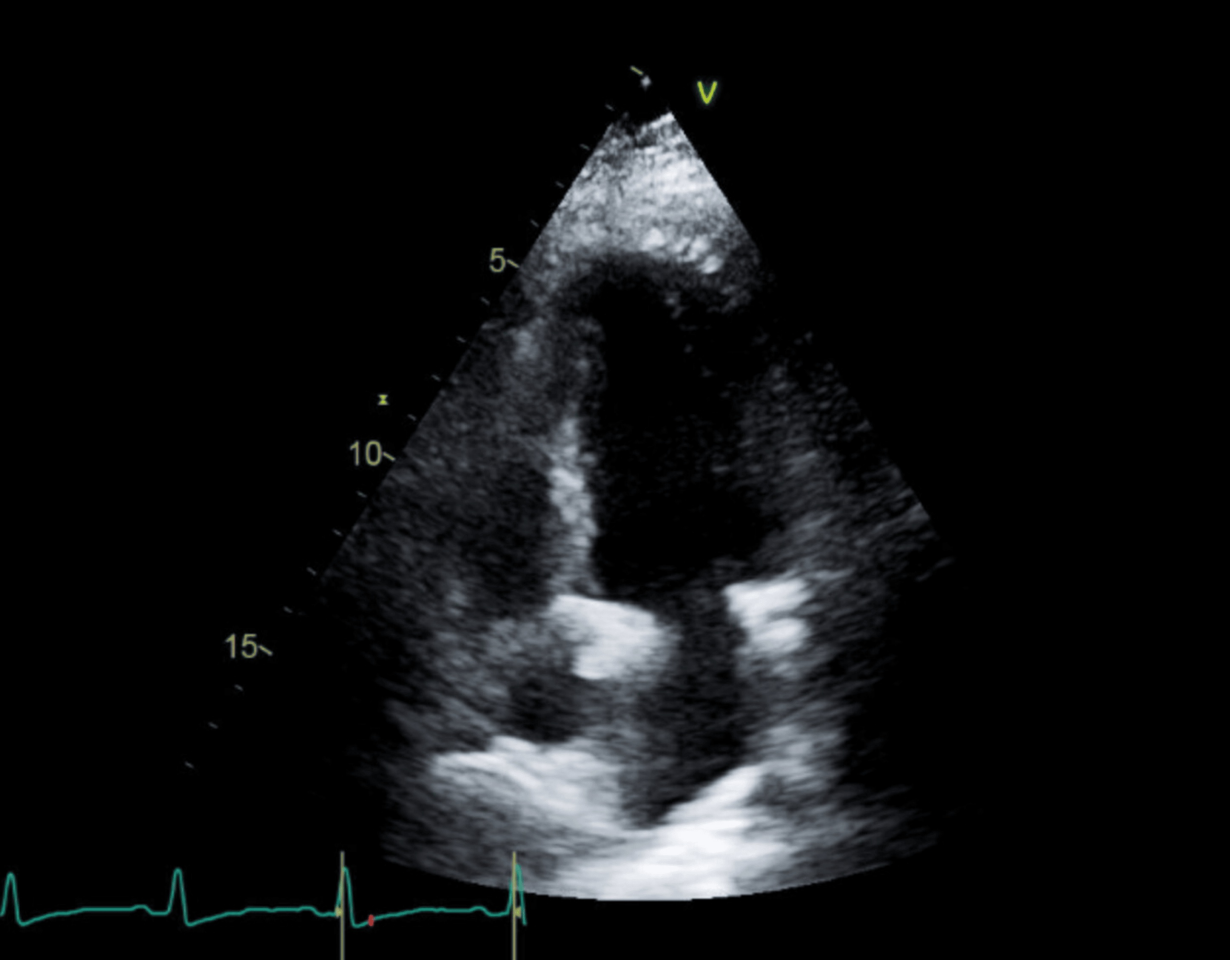
Apical view echocardiogram showing mild mitral valve leaflet thickening and tricuspid regurgitation.

**Figure 5:**
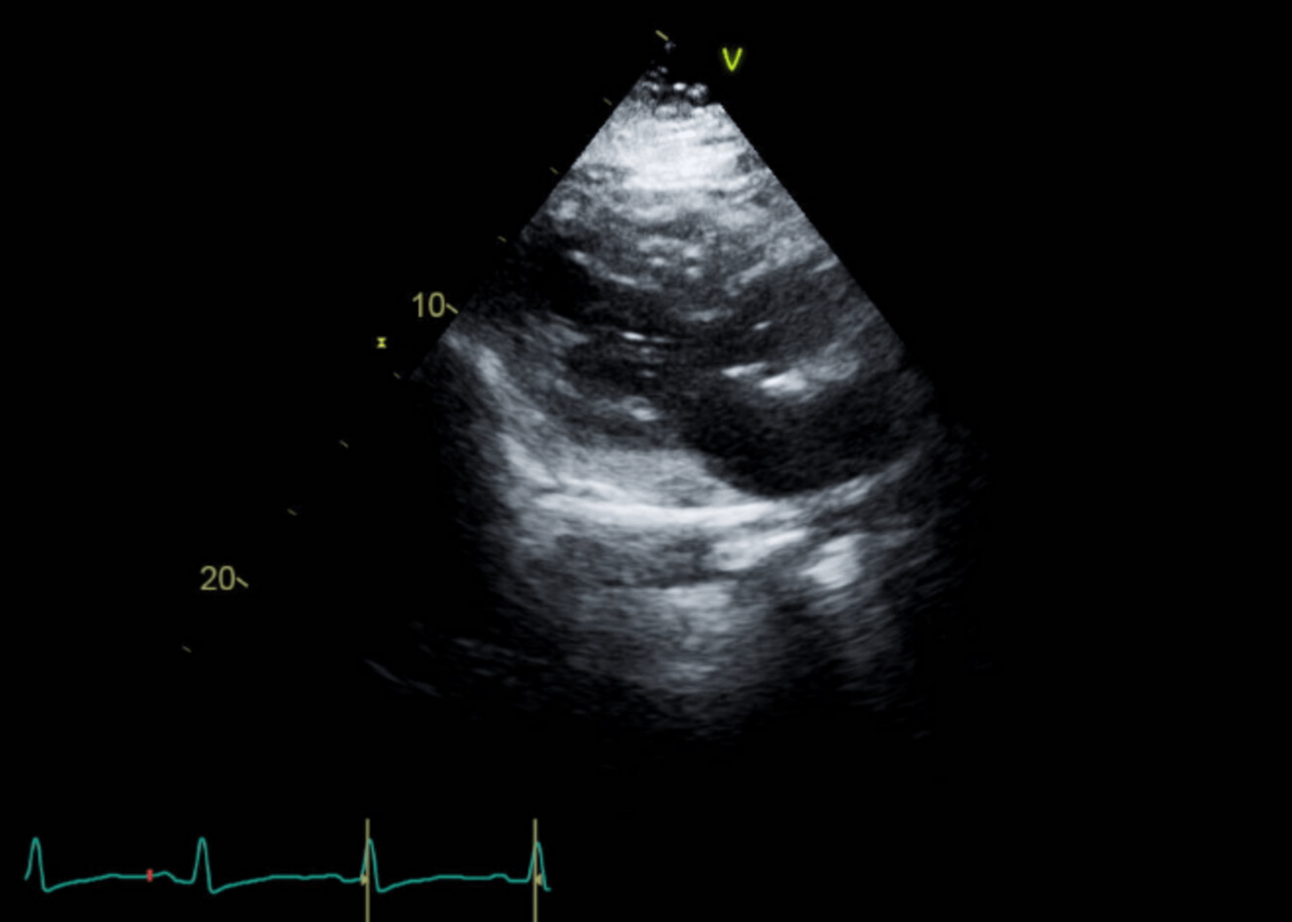
Parasternal long-axis view echocardiogram showing mild mitral valve leaflet thickening and tricuspid regurgitation.

A CTA of the chest demonstrated a 2.6 cm enlarged mediastinal lymph node, which may be reactive in the context of infection or inflammation but was otherwise nonspecific, with mild bibasilar dependent atelectasis, and no pulmonary embolism or evidence of active intrathoracic infection or dissection. A transthoracic echocardiography (TTE) showed an ejection fraction of 65-70%, trivial tricuspid regurgitation, mild thickening of the mitral valve leaflets, which is likely an incidental finding in the absence of mitral stenosis or regurgitation, and no wall motion abnormalities or structural defects (Figures 4, 5). The trivial tricuspid regurgitation is commonly seen in healthy individuals and is not considered hemodynamically significant here.

**Figure 6:**
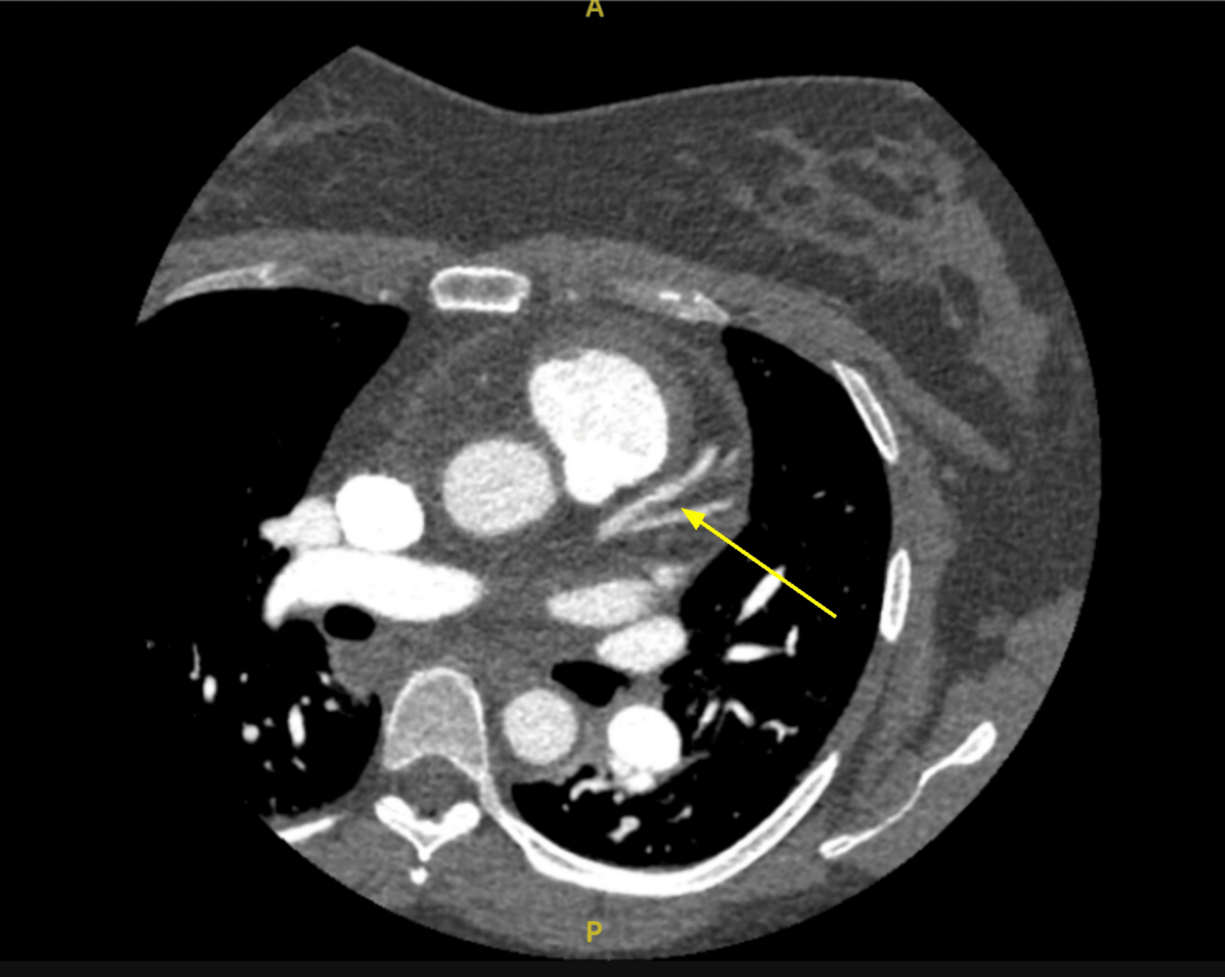
Computed tomography angiography. The yellow arrow points to the intramural hematoma form of spontaneous coronary artery dissection.

Coronary CTA reportedly showed a smooth, 8 mm, well-defined area of mild soft plaque-like narrowing, but this is not the typical appearance of SCAD, which often presents as a radiolucent intramural hematoma or dissection flap (Figure 6). SCAD diagnosis relies heavily on invasive coronary angiography, which was not performed in this case, limiting definitive diagnosis. No additional coronary arteries were noted to be affected on imaging, and no aneurysms or high-grade stenoses were identified. The diagnosis of SCAD came about due to the clinical suspicion based on the patient’s demographic being a young pregnant female, chest pain, troponin elevation, and a lack of atherosclerotic risk factors.

The patient was continued on enoxaparin (Lovenox) for thrombus management and IV ampicillin-sulbactam, later transitioned to outpatient ertapenem via a peripherally inserted central catheter (PICC) line for her Lemierre’s syndrome, and aspirin. Notably, beta-blocker therapy, a cornerstone in SCAD management to reduce shear stress, was initiated with labetalol cautiously to balance blood pressure control with the potential for hemodynamic instability or fetal compromise [19].

The use of dual antiplatelet therapy (DAPT) was not initiated, due to concerns regarding bleeding risk in the setting of concurrent anticoagulation for jugular thrombosis and potential infectious complications. Given the risk of hemorrhagic complications in Lemierre’s syndrome, especially if septic emboli or vascular fragility are present, the decision to avoid DAPT and limit therapy to aspirin and enoxaparin appears judicious [20].

## Discussion

SCAD remains underdiagnosed and underreported, likely due to frequent misdiagnosis or under-recognition, particularly in younger women and during pregnancy. While a female-to-male ratio of approximately 2:1 has been described, studies focusing on pregnancy-associated SCAD suggest an even higher predominance among women, especially in the peripartum period [21]. This case is further complicated by the co-occurrence of Lemierre’s syndrome, a rare and serious condition that rarely overlaps with SCAD.

Pregnancy presents a unique physiological environment that predisposes individuals to both SCAD and Lemierre’s syndrome [1,5,6]. Hormonal fluctuations, increased blood volume and cardiac output, and a hypercoagulable state all contribute to vascular fragility and thrombotic risk [22,23]. While our patient had no known connective tissue disorders, thromboembolic disease, or family history of vascular conditions, her pregnancy, at 22 weeks of gestation, represents a significant contributing factor. She also had no documented pregnancy-related risk factors such as hypertension, preeclampsia, or multiparity [24].

The presentation was highly unusual due to the simultaneous manifestation of two rare vascular conditions in pregnancy. Although arterial complications occur in approximately 8% of Lemierre’s syndrome cases, they typically involve the carotid arteries or cerebrovascular events such as stroke [25]. Coronary artery involvement is exceptionally rare, and to our knowledge, this may represent one of the first documented cases of SCAD occurring concurrently with Lemierre’s syndrome in a pregnant patient.

The diagnosis of Lemierre’s syndrome was based on internal jugular vein thrombosis and clinical presentation. While anticoagulation is not routinely required in uncomplicated Lemierre’s syndrome, it was initiated in this case using enoxaparin (Lovenox), guided by pregnancy-specific considerations and the severity of thrombosis (18). However, the decision to anticoagulate must be balanced against the risk of intramural hematoma propagation in SCAD, particularly during pregnancy. Standard SCAD management recommends discontinuing anticoagulation once the diagnosis is confirmed due to the risk of worsening the dissection [26]. In our case, anticoagulation was continued due to the concurrent Lemierre’s syndrome. Acknowledging the deviation from standard SCAD management, we emphasize that this was a carefully weighed decision made in a multidisciplinary setting. Further studies are needed to guide anticoagulation use when these two rare conditions coexist.

For SCAD, a conservative management strategy was chosen due to the patient’s hemodynamic stability, minimal coronary involvement, and absence of ongoing ischemia. Although percutaneous coronary intervention (PCI) or coronary artery bypass grafting (CABG) may be indicated in unstable or high-risk SCAD, conservative therapy is generally favored when feasible, especially during pregnancy [27]. Beta-blockers are the mainstay of SCAD treatment for their ability to reduce shear stress on the arterial wall [19]. Labetalol was chosen due to its safety profile in pregnancy, despite metoprolol being more commonly used in SCAD management [19]. Low-dose aspirin was continued for its antiplatelet benefits.

Risk factors for SCAD include fibromuscular dysplasia, pregnancy, and hormonal influences such as oral contraceptive use [5,6]. In the context of pregnancy, the mechanism is likely hormonal and structural, rather than thrombotic [9]. Estrogen and progesterone-mediated changes in collagen and arterial wall architecture, combined with the hemodynamic stress of pregnancy, may predispose coronary arteries to dissection. Lemierre’s syndrome, in contrast, is characterized by septic thrombophlebitis of the internal jugular vein, often following oropharyngeal infection [28]. The hypercoagulable state of pregnancy likely contributed to the pathogenesis of both conditions in this case.

## Conclusions

This case illustrates an exceptionally rare overlap of two serious vascular conditions, SCAD and Lemierre’s syndrome, during pregnancy. It highlights the diagnostic and therapeutic challenges in managing vascular complications in this population, particularly when treatment guidelines for one condition may conflict with those for the other. This case underscores the importance of individualized, multidisciplinary care and calls attention to the need for further research into vascular pathology in pregnancy.
